# SG-APSIC1174: High-value instrument management: Rigid endoscopes

**DOI:** 10.1017/ash.2023.111

**Published:** 2023-03-16

**Authors:** Nanthipha Sirijindadirat

**Affiliations:** President, Central Sterilizing Services Association, Bankok, Thailand

## Abstract

**Background:** After 3 years, we discovered that a high-value instrument, rigid endoscopes, needed to be used and cleaned properly. If those instruments are damaged from inappropriate handling or reprocessing, they can pose infection risks to patients. Furthermore, the hospital incurs additional repair costs. **Objectives:** We sought to ensure that specialized instruments are handled and used appropriately in accordance with the manufacturers’ recommendations and that all related units handle and use instruments appropriately. **Methods:** A meeting was convened to establish the purposes and scope of the work, and related data were collected. As a result, we created registration forms for high-risk instruments as well as a survey list for rigid endoscopes. These forms were distributed to appropriate units. We analyzed outcomes, monitored the indicators, and audited work processes. The operating room registered high-value instruments on a form that included instructions for use. A dealer demonstrated how to handle the rigid endoscopes. The CSSD team visited the operating room to emphasize the importance of handling and reprocessing to ensure compliance. The BEM team inspected all endoscopes after use. We created group communication within the software. Using T-DOC, we analyzed and monitored outcomes. We measured the rate of high-risk instrument registration as well as the completeness of registration. We also measured the rate of damage to rigid endoscopes. **Results:** We collected data related to rigid endoscopes and educated the staff to handle and reprocess the instruments appropriately, including instructions for use. High-risk instrument registration forms and surveys were created to record information in the T-DOC system. The staff was invited to educational workshops. The rates of registration, registration completeness, and readiness were measured using the plan–do–check–act (PDCA) method. The results of every indicator reached the expected rates of 100%. **Conclusions:** We achieved our goal of 100% compliance with the new program. All high-value instruments should be registered, and all related staff should be trained to use them appropriately.

